# The FOG‐COA Protocol: Validation of Freezing of Gait Clinical Outcomes Assessments in Parkinson's Disease

**DOI:** 10.1111/ejn.70701

**Published:** 2026-10-17

**Authors:** Erika E. Howe, Pieter Ginis, Kaylena A. Ehgoetz Martens, Moran Gilat, Martina Mancini, J. Lucas Mckay, Jorik Nonnekes, Jennifer Mammen, Jamie L. Adams, David Cella, Alice Nieuwboer, Fay Horak, Simon J. G. Lewis, Jeffrey M. Hausdorff

**Affiliations:** ^1^ Department of Kinesiology and Health Sciences University of Waterloo Waterloo Ontario Canada; ^2^ Clario, Part of Thermo Fisher Scientific Leuven Belgium; ^3^ Department of Rehabilitation Sciences, Neurorehabilitation Research Group (eNRGy) KU Leuven Leuven Belgium; ^4^ Department of Neurology Oregon Health and Science University Portland Oregon USA; ^5^ Department of Biomedical Informatics and Department of Neurology Emory University School of Medicine Atlanta Georgia USA; ^6^ Department of Rehabilitation, Center of Expertise for Parkinson and Movement Disorders, Donders Institute for Brain, Cognition and Behaviour Radboud University Medical Center Nijmegen the Netherlands; ^7^ College of Nursing and Health Sciences University of Massachusetts Dartmouth Dartmouth Massachusetts USA; ^8^ Department of Neurology University of Rochester Rochester New York USA; ^9^ Departments of Medical Social Sciences, Psychiatry, and Neurology Northwestern University Feinberg School of Medicine Chicago Illinois USA; ^10^ Parkinson's Disease Research Clinic Macquarie University Sydney Australia; ^11^ Center for the Study of Movement, Cognition, and Mobility, Neurological Institute Tel Aviv Sourasky University Medical Center—Ichilov Tel Aviv Israel; ^12^ Department of Physical Therapy, Gray Faculty of Medical & Health Sciences, and Sagol School of Neuroscience Tel Aviv University Tel Aviv Israel; ^13^ Rush Alzheimer's Disease Center and Department of Orthopedic Surgery Rush University Medical Center Chicago Illinois USA

**Keywords:** clinical outcomes assessment, freezing of gait, mobility, Parkinson's disease

## Abstract

The development and evaluation of novel treatment options for freezing of gait (FOG) in Parkinson's disease have been limited by an ambiguous definition and lack of standardized clinical outcome assessments (COAs) that 1) reliably assess FOG severity, 2) are responsive to change, and 3) capture patient‐relevant impact. To address these gaps, working groups from the International Consortium on FOG (ICFOG) developed a clinician‐rated outcome—the Giladi Protocol for assessing FOG severity (GP‐FOG), and a patient‐reported outcome—the FOG Quality of Life questionnaire (FOG‐QOL). The FOG‐COA project will evaluate the conceptual framework and psychometric properties of these COAs to determine their fit‐for‐purpose in clinical trials. A total of 105 freezers and 35 non‐freezers from seven international centers will complete the GP‐FOG in the dopaminergic OFF and ON states during two separate visits (7–14 days apart). FOG annotations from video recordings will be used to assess criterion validity. OFF and ON GP‐FOG scores from two trained raters at Visit 1, as well as within‐rater scores across visits, will examine reliability. Responsiveness will be evaluated by comparing effect sizes between OFF and ON GP‐FOG scores. Patient meaningfulness will be assessed through the association between patient‐perceived FOG severity and GP‐FOG scores. Participants will complete the FOG‐QOL at each visit to assess test–retest reliability and validity against the NFOGQ and PDQ‐8. A subset of freezers will undergo cognitive interviews to identify relevant daily life FOG impacts and evaluate FOG‐QOL meaningfulness. Collectively, this work will advance standardized, sensitive, and meaningful FOG‐COAs, supporting regulatory‐grade endpoints and the development of a multi‐dimensional outcome framework.

## Graphical abstract

The international freezing of gait clinical out assessment (FOG‐COA) project aims to validate regulatory‐grade clinician rated and patient rated clinical assessment tools that can be used in clinical trials and advance therapy for FOG in Parkinson's disease.

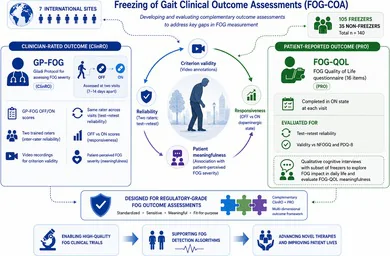

**Abbreviations**
ClinRO: clinician reported outcome
COA: clinical outcomes assessments
DT: dual task
FOG: Freezing of Gait
FOG‐QOL: Freezing of Gait Quality of Life Questionnaire
GP‐FOG: Giladi Protocol for assessing Freezing of Gait Severity
ObsRO: observer rated outcome
PD: Parkinson's disease
PRO: patient reported outcome

## 1 Introduction

Freezing of gait (FOG) is a debilitating symptom that affects upwards of 80% of individuals with Parkinson's disease (PD) and has been reported as one of its most bothersome symptoms (Bloem et al. 2004; Chahine et al. 2020; Nutt et al. 2011). Yet, therapeutic options remain limited, as dopaminergic medications provide only partial relief of FOG for some, while offering no benefit or even exacerbating FOG in others (Amboni et al. 2015; Espay et al. 2012; Lucas McKay et al. 2019; Tosserams et al. 2025). Novel treatment options have been hindered by ambiguity in prior FOG definitions, a lack of standardized clinical measurement tools that reliably evaluate FOG severity and responsiveness to change, as well as the absence of validated instruments that capture the patient perspective on quality of life (Lewis et al. 2022). To address these issues, the International Consortium for Freezing of Gait (www.icfog.org) assembled several workgroups to put forward an updated definition of FOG (Gilat et al. 2026) and to develop novel FOG‐specific clinical outcome assessments (COAs), including a clinician‐rated outcome (ClinRO) and a patient‐reported outcome (PRO) (see Ehgoetz Martens et al. 2026b) based on expert consensus and current guidelines. Together, these new developments provide a comprehensive framework for measuring FOG severity, integrating objective, clinician‐rated, and patient‐reported domains. Although the creation of these new tests is an important step forward, a critical next step is to evaluate the validity, reliability, responsiveness, and meaningfulness of the COAs to determine their suitability for use as endpoints in clinical trials.

The current gold standard performance measure of FOG severity is to calculate the percentage time spent frozen (%FOG) derived from manual video annotations, ideally with consensus across multiple annotators (Cockx et al. 2022; Gilat 2019; Gilat et al. 2026). Video annotations have previously relied on various self‐determined definitions of FOG derived from the definition originally proposed by Nutt et al. (2011), however, the resulting inter‐rater reliability (intraclass correlation coefficient: ICC) across studies has produced a wide range of reliability (%FOG ICC = 0.50 [Scully et al. 2024; ICC = 0.94 Zoetewei et al. 2024]). The prior lack of explicit criteria for determining FOG episode onset and offset leads to inconsistencies in FOG identification and ultimately contributes to variance in %FOG estimates. Additionally, the prior definition did not delineate between different clinical presentations of FOG that have been variably reported in the literature (Gilat 2019; Gilat et al. 2026; Schaafsma et al. 2003; Thompson and Marsden 1995). To address these limitations, an updated FOG definition was recently proposed by Gilat et al. (2026), incorporating both a clinical definition to improve communication as well as a technical definition designed to standardize video annotation based on expert consensus. The technical definition includes explicit criteria for identifying FOG episodes, including delineation of core freezing (where there is cessation of progression) and improved differentiation of FOG manifestations (akinetic, kinetic‐trembling, and kinetic‐no trembling). By reducing uncertainty in episode identification and classification, this updated definition aims to improve consistency in %FOG reporting.

Although video‐based annotation of FOG episodes is considered by many as the gold standard method for quantifying FOG severity, it is often not feasible to apply it as an outcome of FOG severity in widespread clinical or research applications due to its reliance on time‐intensive, offline analysis by trained raters. Emerging approaches using wearable sensors and automated detection algorithms show promise (Gregorčič and Georgiev 2025; Mancini et al. 2025; Salomon et al. 2024), but currently require further optimization, replication, and validation in new cohorts (Mancini et al. 2025), limiting their generalizability and immediate applicability. Cost and infrastructure might also limit their scalability. As such, there remains a critical need for a simple, standardized, and scalable clinical assessment for FOG severity.

The FOG score and the FOG severity test are two existing clinician‐rated outcomes (ClinROs) incorporating common FOG‐provoking conditions, such as gait initiation, turning, and navigating narrow spaces (Scully et al. 2022; Ziegler et al. 2010). Both tests have demonstrated good inter‐rater reliability and validity when evaluated against video‐based registration (Goh et al. 2022; Scully et al. 2024). However, several limitations constrain their utility as standardized clinical outcome measures. First, current scoring approaches lack sufficient granularity, as they do not capture key dimensions of FOG severity such as episode frequency and duration; consequently, brief and prolonged episodes may be scored equivalently (Goh et al. 2022). Second, both tools incorporate the FOG phenotype (i.e., manifestation) as a central component of the scoring scheme despite the lack of current evidence that FOG manifestations have a progressive nature or that festination is a substrate of FOG. In addition, these tools have a concatenated task design and lack defined time constraints, which may result in lengthy tests, particularly for severe FOG and limit the ability to isolate individualized context‐dependent triggers.

To address these limitations, the Giladi Protocol for assessing FOG severity (GP‐FOG) was developed as a standardized, task‐based ClinRO, designed in accordance with the US Food and Drug Administration (FDA) Drug Development Tool qualification guidance, incorporating patient input and a predefined conceptual framework (Ehgoetz Martens et al. 2026a). The GP‐FOG comprises a series of FOG‐provoking conditions and characterizes FOG severity through assessment of episode frequency, duration, and cumulative time spent frozen, based on the updated FOG definition (Ehgoetz Martens et al. 2026a). However, the reliability, validity, and responsiveness to the dopaminergic state of GP‐FOG scores are not yet known.

Although ClinROs are essential for quantifying FOG severity based on clinical observation of the symptom in research and clinical trials, they might not capture the subjective patient experience of FOG or whether changes in its severity translate into meaningful improvements in daily life. Hence, a patient‐reported outcome (PRO) is also critical for evaluating the impact of FOG on daily life and determining meaningful change in clinical trials. Existing self‐report tools, such as the FOG questionnaire (FOGQ), the new freezing of gait questionnaire (NFOGQ), and the characterizing FOG questionnaire (C‐FOG), were developed prior to current regulatory guidance for PRO development. As such, evidence of content validity based on systematic key stakeholder input and predefined conceptual frameworks is lacking (Ehgoetz Martens et al. 2018; Nieuwboer et al. 2009). As a result, these instruments may not fully capture key aspects of the patient's lived experience for FOG. In addition, the NFOGQ demonstrates poor correlation with video‐rated %FOG and lacks sensitivity to change, limiting its suitability as a clinical trial endpoint (Hulzinga et al. 2020). Furthermore, existing self‐report measures primarily assess generalized impacts of FOG on activities of daily living (Nieuwboer et al. 2009), few capture the heterogeneity of FOG triggers or the context‐specific ways in which it affects quality of life (Ehgoetz Martens et al. 2018), and many overlook important psychosocial factors. To address these limitations, a novel PRO, the FOG Quality of Life questionnaire (FOG‐QOL), was developed to capture the physical, emotional, social, and contextual impacts of FOG on daily living (Ehgoetz Martens et al. 2026b). The instrument was designed in accordance with FDA patient‐focused drug development guidance (FDA and CDER 2022; FDA CDER 2022), incorporating patient input and a hypothesized conceptual framework, with a clearly defined context of use in clinical trials (Ehgoetz Martens et al. 2026b). The 16‐item questionnaire assesses the impact of FOG across multiple domains over a four‐week recall period. The next critical step is to evaluate the content validity and psychometric properties of the FOG‐QOL.

Overall, several important approaches have been taken to develop COAs to provide a complementary perspective on FOG severity and its consequences, essential for evaluating meaningful treatment effects in clinical trials from both clinician and patient perspectives. However, comprehensive validation of the GP‐FOG and FOG‐QOL instruments is required to establish their reliability, validity, responsiveness to change, and underlying conceptual structure in accordance with COnsensus‐based Standards for the selection of health Measurement INstruments (COSMIN) standards. In addition, it will be essential to evaluate and verify the conceptual framework that the PRO has adopted through qualitative patient interviews and alongside psychometric performance. Importantly, the PRO and ClinRO instruments are designed to capture complementary aspects of freezing of gait: the GP‐FOG is anchored to the behavioral expression of FOG observed during standardized tasks, whereas the FOG‐QOL is anchored to the subjective patient‐perceived impact of FOG on multiple aspects of daily life. Multiple measurement properties are essential to establish a fit‐for‐purpose COA for regulatory‐grade endpoints.

The primary objective of this study is to evaluate the criterion validity of the GP‐FOG relative to %FOG scored from video using the updated definition. Secondary objectives are to i) evaluate the reliability and validity of the GP‐FOG; ii) determine the responsiveness to change of the GP‐FOG by evaluating participants in the OFF and ON dopaminergic medication states; iii) assess the meaningfulness of the GP‐FOG iv) evaluate the conceptual framework of the FOG‐QOL and relevance to people living with PD; and v) assess the reliability and validity of the FOG‐QOL. This study will employ a multi‐center design to comprehensively evaluate these measurement properties.

## 2 Materials and Methods

The FOG‐COA project is being conducted as an international multi‐center collaborative effort with study sites at Tel Aviv Sourasky Medical Center Ichilov, Israël; Emory University, USA; Radboud University Medical Centre, the Netherlands; KU Leuven, Belgium; Oregon Health & Science University, USA; University of Rochester, University of Massachusetts Dartmouth, USA; Macquarie University, Australia; and University of Waterloo, Canada.

### 2.1 Study Design

This is a prospective, multicenter, observational cohort study with repeated within‐subject assessments designed primarily to evaluate novel in‐clinic clinical and patient‐rated assessments of FOG in PD. Video recordings will be collected to facilitate expert rating of %FOG using a standardized annotation method (Gilat 2019; Gilat et al. 2026). Synchronized, body‐worn sensor recordings will be collected to support future objective analysis of FOG using inertial measurement unit (IMU) data. Free‐living monitoring and a qualitative interview sub‐study are included to support future aims related to symptom characterization and machine learning development.

A total of 140 participants will be recruited across 7 international sites, including 105 participants with self‐reported FOG and 35 participants without self‐reported FOG. Recruitment will be distributed approximately equally across sites. Interested individuals will first undergo prescreening and, if eligible, will provide written informed consent before completing formal screening procedures. Participants who meet all eligibility criteria will then be enrolled. Freezing status will be determined during screening based on the first item of the NFOGQ, which asks whether the participant experienced freezing episodes during the previous month.

Participants initially classified as self‐reported freezers will complete two in‐person assessment visits scheduled 7 to 14 days apart. At each visit, the in‐clinic FOG assessment (GP‐FOG) will be performed first in the practically defined OFF‐medication state following overnight withdrawal of dopaminergic medications and then repeated in the ON‐medication state approximately 1 h after intake of the usual dosage while instrumented with five IMUs (Opals, APDM Clario) located on their lumbar spine, anterior lower leg, and dorsal feet bilaterally (Figure 1). Participants initially classified as non‐freezers will complete one in‐person assessment visit. If freezing is observed during the clinician‐rated assessment at that visit, those participants will be reclassified as freezers and asked to return for a second in‐person visit.

**FIGURE 1:**
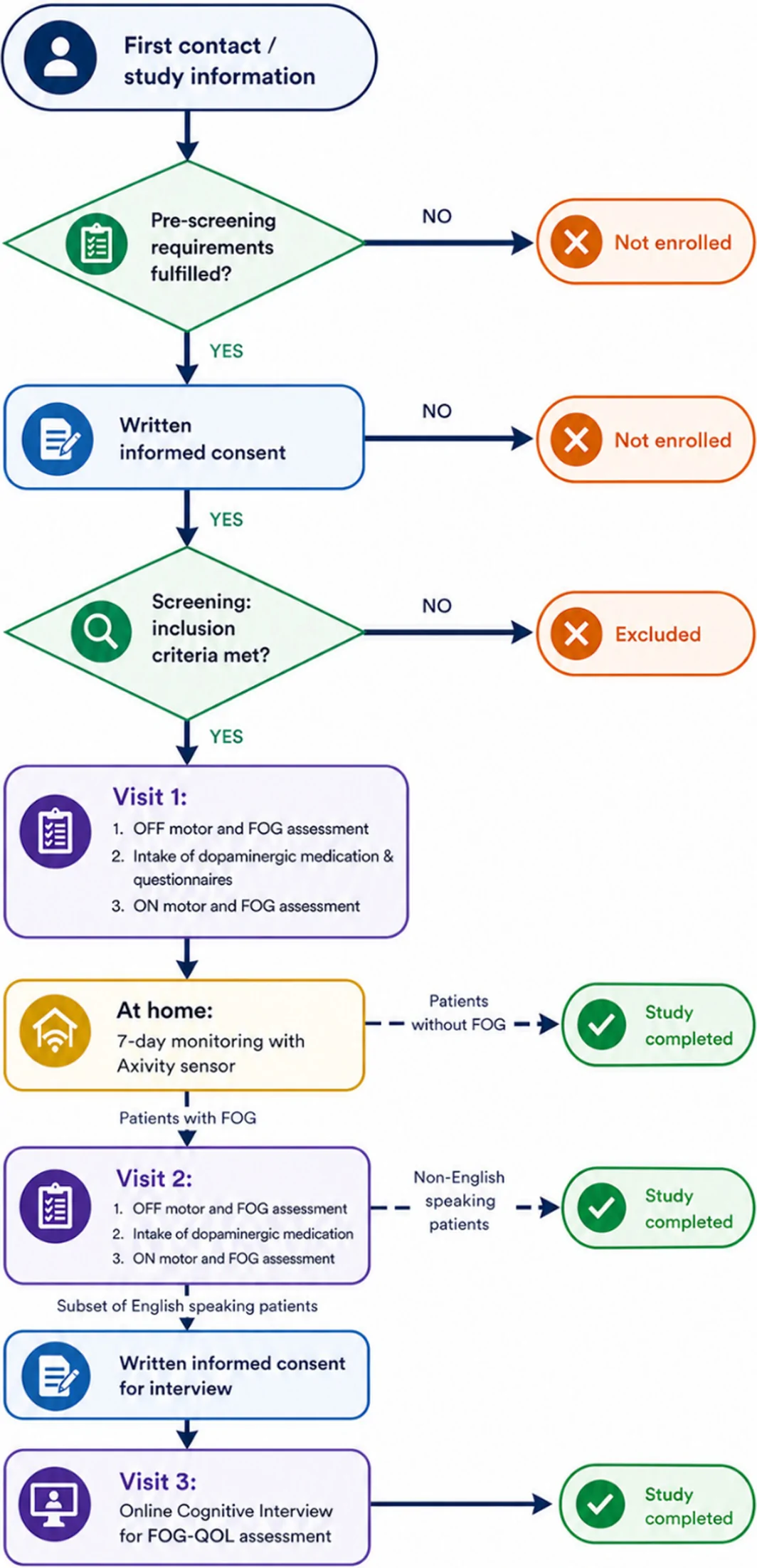
Flowchart illustrating the study procedures for participants with and without freezing of gait (FOG). Visits 1 and 2 are in‐person assessments in both OFF and ON dopaminergic medication states while instrumented with Opal IMU sensors. Cognitive interviews are evaluated the patient reported outcome (FOG‐QOL).

Following Visit 1, all participants will undergo 7 days of home monitoring with an AX6 Axivity inertial measurement unit worn at the level of L5. During the home‐monitoring period, participants will also record dopaminergic medication administration times and sleep–wake periods. These home‐based data will be collected to support secondary analyses and future development of machine learning approaches for freezing of gait characterization. Visit 2 will mirror Visit 1, with in‐clinic assessments including the GP‐FOG conducted in the OFF medication state, followed by the ON state. In addition, a convenience sample of 40 English‐speaking participants with FOG will be invited to participate in a virtual semi‐structured symptom‐mapping interview conducted by trained assessors at the University of Rochester.

### 2.2 Prescreening

Site‐specific initial prescreening will be used to identify candidate participants in accordance with local privacy and ethics requirements. Some sites maintain registries of individuals with PD who have agreed to be contacted about research. Where permitted by local IRB or ethics approval, sites may also conduct limited medical record review under an approved partial HIPAA waiver or similar mechanism.

### 2.3 Screening and Recruitment

Participants with PD will be eligible for the study if they meet all the inclusion and none of the exclusion criteria as outlined in Table 1. Ethical approval has been granted by the local ethics committees at all sites. Written informed consent will be obtained from participants prior to study participation in accordance with the Declaration of Helsinki.

**TABLE 1:** Inclusion and exclusion criteria for the FOG‐COA study.

Inclusion criteria	Exclusion criteria
Able to complete informed consent Age of at least 18 years Able to read and write in the first language of their respective country Diagnosis of idiopathic Parkinson's disease (PD) made by a neurologist according to the Movement Disorders Society guidelines Able to walk independently for 10 m, without walking aid in the ON state No prior functional neurosurgery Stable PD treatment in the 4 weeks prior to and during the study A score of ≥ 1 on the new freezing of gait questionnaire (NFOG‐Q)^a^	Myocardial infarction, hospitalization for unstable angina, stroke, coronary artery bypass graft (CABG), percutaneous coronary intervention (PCI), implantation of a cardiac resynchronization therapy device (CRTD)^b^ Receiving active treatment for cancer or other malignant disease^b^ Uncontrolled congestive heart disease (NYHA class > 3) Acute psychosis or major psychiatric disorders or continued substance abuse^b^ Are diagnosed with other neurological disorder (other than PD) Have orthopedic impairment that significantly impacts gait. Unwilling to temporarily delay anti‐Parkinsonian medication

^a^ Criteria only applicable for self‐identified freezer group.

^b^ Currently or in the past 3 months been hospitalized or treated for NYHA: New York Heart Association.

### 2.4 Assessment Procedures

Participants will arrive for each in‐person study visit in the practically defined OFF state by withdrawing from dopaminergic medication overnight with at least a 12‐h washout period. Motor symptom severity as well as self‐reported measures of both FOG and fall‐related severity are assessed using the Movement Disorders Society‐ Unified Parkinson's Disease Rate Scale part III (MDS‐UPDRS‐III) and self‐report measure using a 10‐point Likert Scale (see Data S1: Patient Meaningfulness Survey). Participants then complete the IMU instrumented GP‐FOG in their OFF state while two independent trained raters score the FOG severity observed during the protocol. Afterwards, participants take their regular dose of dopaminergic medication and complete a series of questionnaires (see below for details). Approximately 1 h after intake of their prescribed dopaminergic medication, when participants are in the ON medication state, the MDS‐UPDRS‐III and GP‐FOG (scored again by the same two independent trained raters) will be repeated along with the Montreal Cognitive Assessment (MoCA) (Table 2). In addition, at Visit 1, companions of the participants with PD will be invited to complete an observer rated outcome (ObsRO) to assess how FOG severity impacts the quality of life of the individual with PD, as well as demographic information, and details of their relationship to the participant. The ObsRO is completed by a companion who is a family member, spouse or close friend of the participant with interaction at least three times per week over the last month to ensure they could effectively report on the participant's FOG severity and impact.

**TABLE 2:** Assessment overview of the FOG‐COA protocol. MDS‐UPDRS (Movement Disorder Society Unified Parkinson's Disease Rating Scale); GP‐FOG (Giladi Protocol for assessing severity of Freezing of Gait); ClinRO (Clinician Rated Outcome); FOG QOL (Freezing of Gait Quality of Life questionnaire); PRO (Patient rated outcome); obsRO (observer rated outcome); NFOGQ (new freezing of gait questionnaire); MoCA (Montreal Cognitive Assessment).

Procedures/assessment	Visits timing (weeks)						
	Screening	Visit 1		Free living	Visit 2^c^		Visit 3^e^
	0–1	1		1–2	2		3–8
Informed consent^a^, ^d^	X						
Inclusion/Exclusion criteria^d^	X						
MDS‐UPDRS‐III^b^		OFF	ON		OFF	ON	
GP‐FOG (ClinRO)^b^, ^f^		OFF	ON		OFF	ON	
Patient Meaningfulness survey^b^		OFF	ON				
FOG QOL (PRO/obsRO^d^)			ON			ON	
NFOGQ			ON			ON	
Dopaminergic medication intake		X			X		
Demographics^d^ and descriptive questionnaires		X					
MoCA		X					
MDS‐UPDRS I, II and IV		X					
Axivity monitoring^f^				X			
Pre‐interview symptom survey							X
Online cognitive interview							X
Concomitant medication		X					X

^a^ Informed Consent must be obtained prior to performing any other Trial‐related procedures.

^b^ Measurement is performed twice during the same visit, once OFF dopaminergic medication, once ON dopaminergic medication.

^c^ Patients without freezing of gait will not have a second visit.

^d^ A companion will only be asked these procedures/assessments.

^e^ For a subset of 40 English‐speaking participants only.

^f^ Instrumented trials with IMU sensors.

### 2.5 The Giladi Protocol for Assessing FOG Severity (ClinRO)

The eight GP‐FOG tasks are standardized across all centers and include single‐ and dual‐task walks, a carrying task, single and dual‐task alternating 360° turns, a box shuffle, a box agility task, and negotiating a doorway (Table 3). For full details, see (Ehgoetz Martens et al. 2026a). The tasks of the GP‐FOG will be video‐recorded (Hero 12, GoPro) for post hoc annotation of FOG.

**TABLE 3:** GP‐FOG task descriptions. Tasks are always performed in this same order. The task setup remains the same for Tasks 1–7 and uses the second setup for Task 8—navigating the doorway.

Standardized GP‐FOG task	Task description
Single‐task walk	From standing, participants perform a 3‐m walk back and forth once, including a 180° turn inside a 50 × 50 cm box outlined with tape on the ground. Upon returning to the starting location, the participant takes a seat in a chair with armrests.
Dual‐task walk	Participants begin a serial subtraction task while standing and, upon hearing an auditory beep, initiate walking toward the box, continuing with the serial subtractions. Once they are inside the box, they turn 180° and return to sit down in a chair with armrests.
Carrying walk	From standing, participants walk to the inside of the marked box on the ground. Once inside the box, participants will make a 90° turn, pick up a size 5 soccer ball resting on a chair, continue turning (making a 180° turn total), and return to a seated position in the chair at the starting location with the ball in hand.
Alternating turns	Participants perform a total of six alternating 360° turns while remaining inside the marked box.
Dual‐task alternating turns	Participants perform a total of six alternating 360°turns while performing serial subtractions from a different randomly assigned number than the dual walking task.
Box shuffle	Participants perform small shuffling steps to trace the outside of the marked box on the floor while trying to remain as close to the box as possible without touching the markings. The participant shuffles around the box in a counterclockwise direction, completes a 180° turn once at the starting location, and then shuffles around the box in the clockwise direction.
Box agility	Participants perform small shuffling steps around the marked box while starting in the forward direction from the same starting location. Instead of tracing the box, participants remain facing the same direction and take leftward, lateral steps followed by backwards steps and then rightward lateral steps.
Doorway	Participants perform a 2.5‐m walk with a doorway (width 90–140 cm) at the 2‐m mark and step into a 50 × 50 cm box marked on the floor. Participants complete a 180° turn inside the box, return through the doorway to the starting position, and finish by completing a 180° turn to finish standing.

### 2.6 Standardized Trial Procedures

All GP‐FOG tasks are performed in a consistent order as quickly and safely as possible, with a maximum trial duration of 60 s. Rest, as needed, is provided between tasks. Patients are kept safe during the trial by the primary rater walking to the side and slightly behind them. The rater is taught not to touch the subject except to avoid a fall. Standardized guarding instructions and training across centers are outlined in the GP‐FOG manual (Ehgoetz Martens et al. 2026a). Trial onset is standardized with an auditory beep generated by the commercial Mobility Lab V2R software. All trials begin and end with this auditory tone. In the walking and carrying tasks, participants are instructed to start “anytime” after the beep to allow for the occurrence of self‐initiated gait failure (Amundsen Huffmaster et al. 2020); in all other tasks, they begin at the beep. Dual‐task conditions require serial subtraction by 3 s from a different random 3‐digit starting number in each trial. Participants begin subtracting aloud before movement initiation and will be allowed to practice briefly while seated before testing.

### 2.7 Quality Assurance

Prior to the recruitment of participants, raters at each of the seven centers will complete an online training for rating of the GP‐FOG ClinRO as well as separate training for annotating FOG using the updated technical definition to ensure their comprehension (Gilat et al. 2026). Training will consist of an online synchronous and asynchronous tutorial. Once the training is completed, raters/annotators will be required to complete an online certification prior to initiating data collection. If the same individual is trained in both the ClinRO scoring and annotation, they will be required to complete a minimum of five ClinRO collections prior to completing annotation training. To further support implementation, regular (monthly) meetings will be held to address protocol inquiries and optimize consistency between centers.

### 2.8 In‐Person Scoring

The GP‐FOG is scored in real‐time by two independent and trained raters in both the OFF and ON states at Visit 1, and again by the same primary rater in Visit 2 in the OFF and ON states, one week later. Consistent primary raters are necessary to evaluate test–retest reliability. Raters are blinded to each other's scoring and instructed not to discuss scoring. For each task, there are two dimensions of scoring (Table 4): FOG frequency and cumulative FOG duration. Scores for each dimension range from 0 (no freezing observed) to 4 (severe freezing or unable to complete the task due to FOG). The FOG frequency score is delineated by both the number of freezing episodes observed within the task (single versus many), but also the approximate duration(s) of the episode(s) (brief: ≤ 1 s, or long: > 1 s). Additional scoring instructions can be found in the GP‐FOG manual (Ehgoetz Martens et al. 2026a). The cumulative FOG duration score is a %FOG estimate based on the duration of the task and the proportion of the task spent frozen. Each task has a total possible score of eight between the two score dimensions for a total maximum score of 64. If a participant is unable to complete the task primarily due to FOG, a score of four on both dimensions is given. Task incompletion could either be due to exceeding the 60‐s time limit or researcher intervention to prevent a fall that was triggered by FOG. The number of completed turns will also be recorded for each alternating turn task. In addition to each GP‐FOG score, raters will evaluate their confidence using 0 = not confident, 1 = somewhat confident or 2 = confident.

**TABLE 4:** GP‐FOG scoring scheme. FOG frequency sub‐score (/32) and cumulative duration sub‐score (/32) and total GP‐FOG scores (/64).

	0 (None)	1 (Mild)	2 (Moderate)	3 (Severe)	4 (Unable)	Sub score total
FOG frequency	0 = no freezing	1 brief FOG episode (lasting ≤ 1 s)	Many brief FOG episodes (> 1 episode) OR 1 long‐lasting episode (lasting ≥ 2 s)	Many long FOG episodes (> 1 episode)	Unable/assistance required. Note: If a trial exceeds a maximum of 60s due to FOG	/32
Cumulative duration of FOG		< 10% trial	> 10% and < 50% trial	≥ 50% trial		/32
Total	/64					

### 2.9 Video Annotations: Percentage Time Frozen

The GP‐FOG video‐recordings are standardized across centers to capture the participants' full body in both the frontal and sagittal planes (Figure 2). In situations where two‐camera capture is not possible, the frontal plane is prioritized. Video recordings are synced with the IMU capture software (Mobility Lab V2R, Clario Inc.) using a custom‐built sync app such that both frontal and sagittal video recordings begin 3 s prior to the start of the Opal recording to capture quiet stance and end ~2 s after the task is complete or timed out. All videos will be pseudonymized through coding and blurring of the faces. The footage will be downsampled from 60 to 30 frames per second (fps) and stripped of audio for anonymity. Processed videos across all centers will be uploaded to a central data repository (ManGO of KU Leuven, Belgium), whereafter they are randomized and redistributed to the other centers to be annotated for FOG episodes by trained annotators. Annotators are thereby a different individual from the raters scoring the GP‐FOG for that participant. Annotators will also be blinded to medication state, visit, and group to minimize bias. Annotations are based on the updated technical definition of FOG (Gilat et al. 2026) using the annotation software ELAN (Gilat 2019). GP‐FOG task scores will be aggregated to participant‐level summary scores for primary analyses to avoid non‐independence across tasks within individuals. The cumulative %FOG is calculated by dividing the total time spent frozen across all GP‐FOG tasks by the total duration required to complete those tasks, as shown in the equation below. Item %FOG will also be calculated and used in exploratory analyses to examine task‐level variability using mixed‐effects models with participant as a random effect. In the case of a task that is incomplete due to a near‐fall, intervention to prevent a fall, or a participant's inability to safely continue, %FOG will be calculated using the observed portion of the task. However, these trials will remain noted for the reason of termination, and further sensitivity analyses will be performed by excluding tasks and/or participants with early‐terminated trials.

$$\text{Cumulative}\%\mathrm{FOG}=\frac{\sum_{i}^{n\;}\;{\mathrm{TF}}_{i}}{\sum_{i}^{n}\;D_{i.}}$$

**FIGURE 2:**
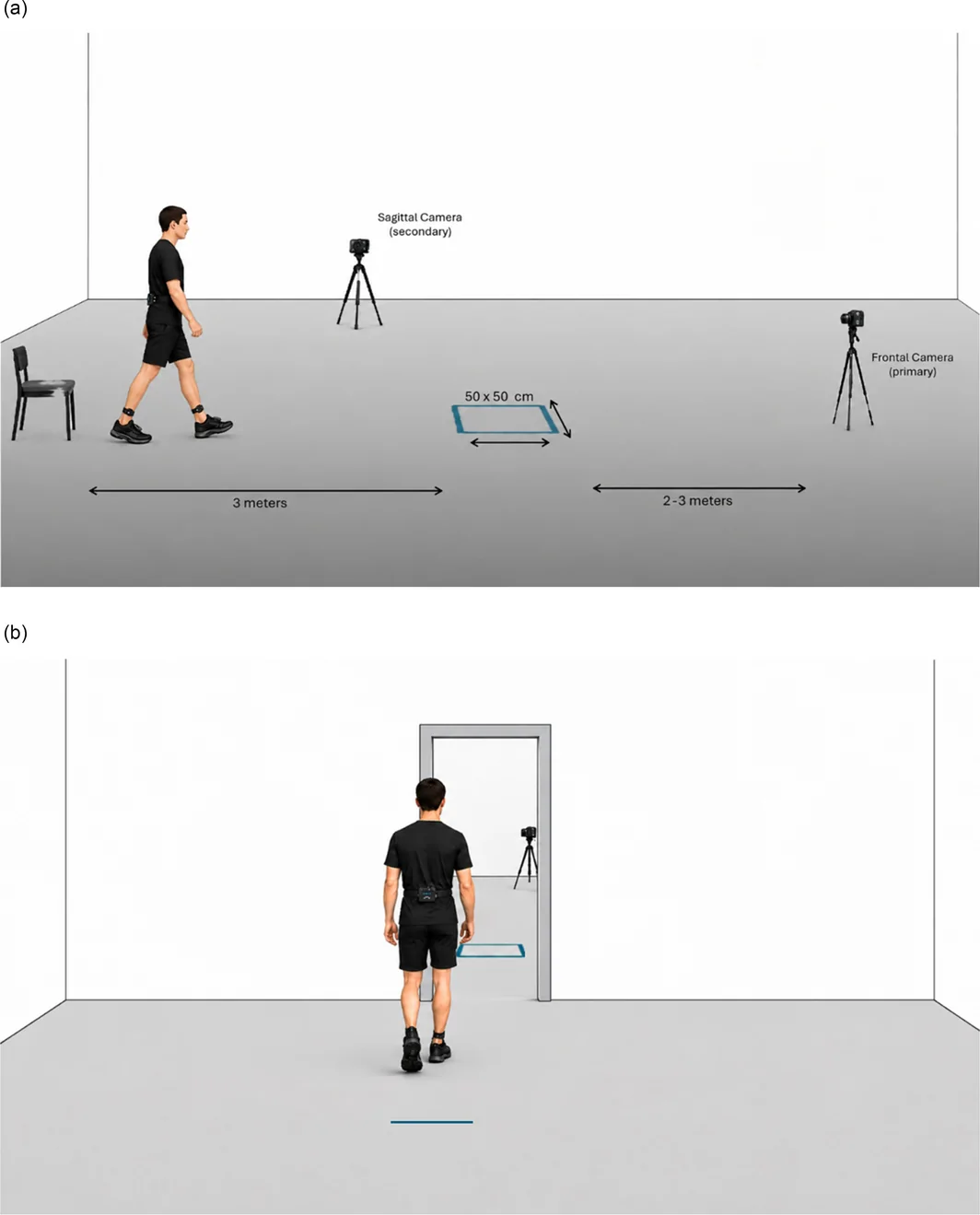
GP‐FOG Task Set up. Panel on the top is the setup for tasks 1 to 7 while the panel on the bottom is the setup for task 8 through the doorway.

TF_i_ is the time frozen in the task; D_i_ is the duration of the task; *n* = number of tasks.

### 2.10 Patient Meaningfulness Survey

Patient meaningfulness of the GP‐FOG is evaluated using a self‐reported survey that utilizes a 10‐point Likert scale to gain patients' perception on both task‐level and ClinRO‐level FOG severity (see Data S1: Patient Meaningfulness Survey). After each GP‐FOG task, participants will be asked to report on the task difficulty, their FOG severity, the test's ability to provoke their FOG, as well as the task's relevance to daily life using a 10‐point Likert scale. Moreover, at the end of OFF and ON state ClinROs, participants will be asked to rate their overall FOG severity, as well as the test's ability to provoke their FOG using a 10‐point Likert scale, and their perceived change in FOG severity between OFF and ON (Mammen et al. 2023a, 2023b). Additional open‐ended questions will allow participants to share how the GP‐FOG tasks relate to daily activities that provoke their FOG, along with any FOG‐provoking activities that are not similar to the GP‐FOG.

### 2.11 The FOG Quality of Life Questionnaire (PRO)

The 16‐item FOG‐QOL questionnaire asks participants to evaluate how FOG affects different aspects of daily life over the past 4‐week period irrespective of medication state. Items were developed to capture key domains: mobility (e.g., physical function, walking, turning, and fall risk), emotional well‐being (e.g., feelings, confidence, self‐worth), social participation (e.g., leisure activities, social relationships, family), contextual challenges (e.g., multi‐tasking, navigating public spaces), and overall quality of life. Development of the FOG‐QOL with a patient advisory committee and its conceptual framework is described elsewhere (Ehgoetz Martens et al. 2026b). The items are anchored to a 5‐point Likert scale ranging from 0 (“never”) to 4 (“almost always”). Participants will complete the FOG‐QOL in the ON state at both study visits. Given prior evidence of discordance between patient and caregiver perceptions of FOG severity (Nieuwboer et al. 2009), a companion‐reported (FOG‐QOL ObsRO) version will be administered at the first visit when a care partner is available.

### 2.12 Participant Descriptors

Within the waiting period after intake of dopaminergic medication on Visit 1, participants will complete a series of clinical questionnaires (descriptors) and patient‐reported outcome measures to capture relevant psychological, behavioral, and PD and FOG‐related characteristics (Table 5).

**TABLE 5:** Participant descriptors.

Descriptors	
PD severity	Movement Disorder Society‐Unified Parkinson's Disease Rating Scale (MDS‐UPDRS Parts I—IV) (Goetz et al. 2008)
FOG subtype	Characterizing Freezing of Gait Questionnaire (C‐FOG) (Ehgoetz Martens et al. 2018)
Cognitive function	Montreal Cognitive Assessment (MoCA) (Nasreddine et al. 2005)
Anxiety/depression	Parkinson's Anxiety Scale (PAS) (Leentjens et al. 2014)
	Geriatric Depression Scale (GDS‐15) (Sheikh and Yesavage 1986)
Mobility and falls	Falls Efficacy Scale International (FES‐I) (Yardley et al. 2005)
	Fall History
Quality of life	Parkinson's disease questionnaire 8‐item (PDQ‐8) (Jenkinson et al. 1997)
Sleep	REM sleep behavior disorder screening questionnaire (RBDSQ) (Stiasny‐Kolster et al. 2007)
Medication	Levodopa equivalent daily dose (LEDD) (Jost et al. 2023)
Other	Demographics^a^

Abbreviation: PD: Parkinson's disease.

^a^ Demographic information included age, sex, race/ethnicity, gender identity, sexual orientation, marital status, living arrangements, education, employment, height, weight, smoking history, alcohol consumption, self‐rated health condition, and disease duration.

### 2.13 Free‐Living Monitoring

At the end of the first study visit, both freezer and non‐freezer participants are outfitted with an Axivity IMU sensor on their lumbar spine (AX6, water‐resistant, 11 g, 33 mm × 23 mm × 9 mm) and instructed to wear it continuously for 7 days to record movement in their free‐living environments while they perform their normal routine daily‐life activities. In addition to wearing the IMU sensor, participants will record medication intake times as well as timing in and out of bed (Data S2: Activity Diary).

### 2.14 FOG‐QOL Symptom Mapping Interview

In a subset of English‐speaking participants with FOG (*N* = 40), semi‐structured cognitive interviews will be conducted to evaluate the content validity and conceptual framework of the FOG‐QOL and to understand how FOG affects a patient's perceived QOL. In preparation for the online interviews, participants will be asked to complete a symptom survey that is a binary (yes/no) response to a comprehensive list of PD‐related symptoms experienced (Data S3: Pre‐Interview Survey). Interviews will be conducted virtually by trained qualitative researchers within 6 weeks after the second study visit and in their ON medication state. The interview consists of three components: 1) symptom mapping to characterize each participant's experience of FOG, 2) cognitive debriefing of FOG‐QOL items to assess comprehension, relevance, and completeness, and 3) mapping of the FOG‐QOL items to participant‐reported symptoms and impacts. Symptom mapping is a structured qualitative technique in which participants and researchers co‐develop a visual representation of symptoms and their relative importance/bothersomeness (Mammen et al. 2023a, 2023b). Interviews will be audio‐recorded, transcribed verbatim, and analyzed using a combination of content analysis and thematic analysis. Coding will be generated iteratively alongside data collection (Ehgoetz Martens et al. 2026b).

### 2.15 Statistics

#### 2.15.1 Sample Size Calculation

Previous research reported *r* = 0.72 between the Ziegler FOG score and %FOG (Goh et al. 2022). Because the GP‐FOG was developed to address limitations of existing clinical FOG measures, we hypothesized that it would demonstrate stronger criterion validity with %FOG. For sample size estimation, a target correlation of *r* = 0.82 was specified, representing an absolute increase of 0.10 over the previously reported estimate. With 80% power and an alpha = 5%, 101 freezers are required to detect such a degree of criterion validity. For reliability, the power analysis was also based on intra‐rater (ICC_2,1_ = 0.80) and test–retest (ICC_3,1_ = 0.91) reliability from the Ziegler test (Goh et al. 2022). Since the sample studied had only mild FOG and the study design had limitations, these ICCs may be inflated. Thus, with a targeted ICC of 0.85, 80% power, and an alpha = 5%, 101 freezers are also required to achieve intra‐rater reliability. As such, we set our recruitment target to 105 PD patients with FOG, which accounts for marginal dropout, given the primary outcome is based on the Visit 1 data. It is noteworthy that this sample size will also adequately power our additional analyses of interest on inter‐rater reliability, test–retest reliability, responsiveness to dopaminergic medication and construct validity requires between 99–101 patients (Borg et al. 2022). A convenient sample of *N* = 5 non‐freezers at each center (*N* = 35) will be included as a comparative group to evaluate known group validity.

#### 2.15.2 Planned Analysis

The primary analysis of interest will evaluate the criterion validity of the total GP‐FOG score relative to the accumulated %FOG across all GP‐FOG tasks in OFF at Visit 1. Other criterion validity outcome measures of interest include the ON state GP‐FOG scores, as well as examining the sub‐scores (FOG frequency and cumulative duration) in both medication states. Secondary analyses will further assess reliability and construct validity in OFF and ON separately, and medication responsiveness (OFF versus ON) as outlined below. Reliability and validity analyses will be conducted in the freezer group, whereas the data from non‐freezers will be used for known‐group validity analyses. All analyses will be conducted using R (RStudio v 2024.04.2, Vienna, Austria) and statistical significance will be assessed using two‐sided tests with *α* = 0.05. Descriptive statistics will be presented as means and standard deviations for normally distributed variables, or medians and interquartile ranges for skewed and ordinal data, and frequencies for categorical data.

Analyses will be performed separately for OFF and ON medication states, beyond responsiveness tests. Given the expected non‐normal and potentially zero‐inflated distribution of FOG‐related measures, normality will be assessed using visual inspection and Shapiro–Wilk tests. Nonparametric methods (e.g., Spearman correlations, Mann–Whitney *U* tests) will be used where appropriate. In addition, distributional characteristics (e.g., skewness, kurtosis) will be reported to aid interpretation. For construct, convergent, and divergent validity measures, a Pearson correlation will be used if the data are normally distributed or a Spearman correlation if the data are skewed. ICC values will be interpreted using established thresholds (e.g., < 0.5 poor, 0.5–0.7 moderate, 0.7–0.9 good, > 0.9 excellent reliability). Missing data will be assessed for patterns and handled using either complete‐case analysis or appropriate imputation methods, depending on the extent and mechanism of missingness. The extent and patterns of missing data will be examined. If missingness exceeds 10%, multiple imputation methods will be considered under a missing‐at‐random assumption. Sensitivity analyses will compare imputed and complete‐case results.

Criterion validity, using %FOG as the reference standard, will be assessed by examining the association between GP‐FOG scores and cumulative %FOG derived from expert video annotations. Correlation coefficients will be calculated between GP‐FOG total scores and %FOG. Additional analyses will examine correlations between %FOG and the GP‐FOG sub‐scores (FOG frequency and cumulative FOG duration). Criterion validity will be supported by moderate correlations (*r* ≥ 0.40) between GP‐FOG scores and the reference standard %FOG. Agreement between GP‐FOG scores and %FOG will also be explored using Bland–Altman plots. GP‐FOG task scores will be aggregated to participant‐level summary scores for primary analyses to avoid non‐independence across tasks within individuals. Exploratory analyses may examine task‐level variability using mixed‐effects models with participant as a random effect.

Inter‐rater reliability of the GP‐FOG will be assessed by comparing the total GP‐FOG score (and the two sub‐scores) assigned independently by two raters. Reliability will be quantified using ICCs (2,1) based on a two‐way random effects model for absolute agreement. Inter‐rater reliability will be supported if total scores by different raters show high consistency (ICC > 0.7), indicating that the measure can be reliably administered across trained assessors. To account for potential between‐center variability, exploratory analyses will assess center effects using mixed‐effects models with site included as a random effect. If significant center effects are observed, sensitivity analyses will be conducted, adjusting for center.

Test–retest reliability will be assessed by comparing the total GP‐FOG score (and the two sub‐scores) across Visits 1 and 2, measured by ICCs (3,1). Measurement error will also be estimated by calculating the minimal detectable change (MDC). Test–retest reliability will be supported if GP‐FOG total and sub scores remain stable across visits (ICC > 0.7), indicating that the measure yields reproducible results when clinical status is expected to be unchanged.

Construct validity of GP‐FOG will be evaluated by examining convergent and divergent validity and known group validity analyses with established measures. Convergent validity will be assessed using Pearson or Spearman correlations between GP‐FOG scores and the NFOG‐Q total score. Convergent validity will be supported if a moderate or stronger association is observed (*r* ≥ 0.40). Divergent validity will be evaluated by examining associations between GP‐FOG scores and the tremor sub‐score of the MDS‐UPDRS‐III. Divergent validity will be supported if this association is weak (*r* < 0.40). Known‐group validity will be examined by comparing GP‐FOG scores between participants classified as freezers and non‐freezers based on item 1 of the NFOGQ. Group differences will be evaluated using the Mann–Whitney U test due to the expected non‐normal distribution of scores and floor effects among non‐freezers. Receiver operating characteristic (ROC) curve analyses will be used to evaluate the ability of GP‐FOG scores to discriminate between freezer status. The area under the curve (AUC) will be reported as a measure of classification performance. In addition, the proportion of correctly classified participants will be calculated. Higher AUC values and a greater proportion of correct classifications will indicate stronger known group validity. Floor and ceiling effects will be evaluated by calculating the proportion of participants achieving the minimum and maximum possible scores for GP‐FOG and FOG‐QOL. These effects will be considered present if more than 15% of participants achieve the lowest or highest possible scores.

Responsiveness of the GP‐FOG ClinRO to medication state will be assessed by examining the magnitude of change in GP‐FOG total scores between the OFF and ON dopaminergic medication states. Standardized response means (SRM) will be calculated to quantify the effect size. Associations between GP‐FOG change scores and changes in %FOG, as well as self‐reported FOG severity changes, will also be evaluated using correlations. Larger effect sizes and stronger associations with objective and patient‐reported change will indicate greater responsiveness of the GP‐FOG.

Meaningfulness and patient perceptions of GP‐FOG will be assessed using the Patient Meaningfulness Survey (PMS), which captures domains of relevance, symptom burden, and perceived FOG severity. Associations between GP‐FOG total score (and sub‐scores) for clinically rated FOG severity and PMS responses of patient‐reported FOG severity will be examined in both OFF and ON medication states to assess patient meaningfulness of the overall assessment. Furthermore, change score of the GP‐FOG from OFF to ON and the PMS perceived FOG severity change with medication association will be examined to evaluate what ClinRO score indicates a meaningful change in the patient's FOG severity. In addition, item‐level analyses will be conducted in the OFF state to further examine the meaningfulness of individual GP‐FOG tasks, addressing perceived freezing severity within each task, task difficulty, relevance for provoking FOG, and similarity to real‐world activities. Stronger positive correlations and coherent score patterns across PMS response levels will indicate meaningfully reflected patient‐experienced FOG severity and impact.

To complement quantitative validation, qualitative analysis will be conducted to evaluate the content validity and conceptual framework of the FOG‐QOL. Interview transcripts and symptom maps will be analyzed using a combination of content analysis and thematic analysis (Mammen et al. 2023a, 2023b). Content analysis will be used to identify and categorize symptoms and their impacts, including their frequency and perceived importance. Thematic analysis will be used to identify important or recurring ideas, common experiences, and key concepts related to symptoms and their impact on daily life. Frequency and percentage of participant agreement (i.e., consensus) will be assessed on four key criteria, including whether the item is: (1) understandable, (2) personally relevant to current experiences, (3) uses appropriate answer options, and (4) useful to measure progression of FOG. Consensus will be compared for each item by criteria using a minimum threshold of ≥ 50%. Items with less than 70% consensus will be evaluated using valence coding to qualitatively assess areas for improvement.

To assess the test–retest reliability of the FOG‐QOL, FOG‐QOL scores between Visits 1 and 2 will be compared and quantified using two‐way random effects for absolute agreement ICCs (ICC (3,1)) to demonstrate stable reporting between visits (ICC > 0.7). Construct validity of FOG‐QOL will be examined by assessing associations between PRO scores and disease‐specific and FOG‐specific measures reflecting impact and quality of life. Spearman or Pearson correlation coefficients will be calculated between the FOG‐QOL total score and the NFOG‐Q scores, PDQ‐8 total, and mobility and related subdomains. Moderate to strong associations (*r* ≥ 0.40) will be interpreted as supporting evidence for construct validity.

Additional analyses will be conducted to explore the structural validity and internal consistency of the GP‐FOG and FOG‐QOL. The underlying dimensional structure of each tool will be examined using principal component analyses with varimax rotation to identify latent components that represent shared variance across individual task scores or item responses. Dimensionality will be supported if tasks or items cluster onto interpretable components and are consistent with the hypothesized conceptual framework of FOG. Criteria for component retention will include eigenvalues > 1, inspection of scree plots, and interpretability of factor loadings. Rotated factor loadings will be examined to determine whether tasks or items cluster into coherent dimensions reflecting distinct aspects of FOG. The proportion of variance explained by each factor will be reported to characterize the structural composition of each COA. Internal consistency will be quantified for each identified domain using Cronbach's alpha. Internal consistency will be supported if items within each domain demonstrate strong interrelatedness, indicating that the ClinRO/PRO reliably capture the intended constructs.

## 3 Discussion

This study aims to validate two newly developed FOG severity COAs from both clinician‐rated and patient‐reported perspectives using objective measures of %FOG based on a recently updated FOG definition and standardized annotation procedures (Gilat et al. 2026), as well as cognitive interviews to identify patient meaningful change, impact, and relevance in daily life. The clinician‐rated outcome (GP‐FOG) was developed to measure observable FOG severity using real‐time clinical scores, whereas the patient‐reported outcome (FOG‐QOL) was designed to examine the impact of FOG on an individual's daily living and quality of life. Together, these FOG‐COAs evaluate complementary dimensions of FOG, with the GP‐FOG anchored to observable behavior and the FOG‐QOL providing patient‐perceived impact. This approach supports a more comprehensive characterization of FOG severity and represents an initial step toward harmonized, regulatory‐relevant outcome measures in PD.

FOG is notoriously difficult to provoke in the clinical and research setting (Giladi and Nieuwboer 2008; Nonnekes et al. 2014). Currently available clinician tools either rely on patient recall, using self‐reported measures such as the NFOGQ, C‐FOG, or use of the single‐item on the MDS‐UPDRS‐III that evaluates FOG severity, a likely insensitive task (Barthel et al. 2016). Others have developed several FOG‐provoking assessment tools (Ziegler et al. 2010; Scully et al. 2023), but these tools and their scoring schemes either lack specificity to FOG frequency, duration or number of episodes, or have unknown responsiveness to medication and meaningfulness to patients.

The proposed study has several notable strengths. The experimental protocol to evaluate the GP‐FOG and FOG‐QOL represents, to our knowledge, the largest multi‐center dataset for the assessment of FOG severity to date. Although previous tools have demonstrated reliability and validity, these studies have typically been limited to relatively small sample sizes that have not been tested in both the ON and OFF levodopa states (Goh et al. 2022; Scully et al. 2024). In contrast, the present work will include 105 PD patients with FOG and 35 PD patients without FOG across seven international centers and will be delivered in multiple languages (English, Dutch, and Hebrew), thereby enhancing the generalizability of findings. Moreover, testing the same patients in both OFF and ON levodopa states provides a measure of sensitivity to change. The assessment of the tool's responsiveness to dopaminergic therapy will support its utility for tracking treatment effects. Another key strength is the composite assessment of ClinRO and PRO outcomes. This aligns with growing recognition that FOG is best characterized across complementary measurement domains rather than a single outcome type (Lewis et al. 2022; Proietti et al. 2025). Moreover, this approach allows for evaluation of whether these instruments capture overlapping or distinct constructs, as well as the extent to which the GP‐FOG aligns with the FOG‐QOL's perceived impact and meaningfulness in daily life. Adding an interview of subjects after testing provides important information about how the patients perceive FOG as affecting their daily lives and how the GP‐FOG tasks reflect how FOG impacts their mobility. In addition, asking care‐partners about the effect of their partner's FOG on QOL (ObsRO) enhances validity as FOG may be under‐recognized or inaccurately recalled by patients. Lastly, the concurrent data collection with IMUs during the GP‐FOG, with its precise synchronization with video, will be a source for external validation of existing automatic algorithms for FOG detection.

Importantly, the development and evaluation of the FOG‐QOL have been guided by established methodological standards for outcome measurement, including alignment with the COSMIN framework (Mokkink et al. 2019). This approach ensures a rigorous and transparent evaluation of measurement properties, integrating both qualitative (e.g., cognitive interviews) and quantitative (e.g., psychometric testing) methods to support content validity, structural validity, and reliability. Adherence to such frameworks strengthens the interpretability and credibility of the PRO and aligns with expectations from regulatory bodies, such as the U.S. FDA, for the development of fit‐for‐purpose COAs. Furthermore, the protocol and rating schemes were designed with clinical trial operationalization in mind and, therefore, also incorporated multiple raters (inter‐rater reliability based on real‐time scoring), test–retest reliability within a rater anchored to video annotation as the benchmark within the study design. That, combined with the simultaneous construct and divergent validity efforts, holds potential to make a significant leap forward on FOG assessment that can be used as clinical trial endpoints.

Rigorous standardization procedures, training, and certification of test administrators will be implemented to ensure consistency across testing centers. All raters administering the GP‐FOG will undergo structured training and certification, requiring agreement within 5% of expert consensus ratings. Similarly, annotators responsible for deriving the %FOG from video recordings must complete standardized training and achieve an overlapping consensus relative to expert annotations. Having these training certifications employed helps ensure consistent inter‐rater agreement when scoring FOG from both clinician‐rated and video annotation %FOG perspectives. Finally, the scale and standardization of this study provide an opportunity to develop a centralized library of annotated assessments that can support future development of training and certification modules, ultimately facilitating broader dissemination and implementation of standardized FOG assessment protocols.

Several limitations should be acknowledged. First, assessments in the ON medication state will be exclusively conducted following the OFF state within the same visit. Although this approach is being selected to enhance feasibility and reduce participant burden by minimizing the number of study visits, it may consequently influence estimates of responsiveness to medication. However, criterion validity and reliability are evaluated in the practically defined OFF state and are therefore unaffected by assessment order. To assess the potential influence of learning effects, responsiveness will be examined at both study visits, and consistency of responsiveness estimates across visits will be evaluated. Second, the qualitative component of the study, including the cognitive interviews used to evaluate the content validity and conceptual framework of the FOG‐QOL, is limited to English‐speaking participants. As such, further work will be required to confirm that the conceptual framework and item relevance are congruent across different languages and cultural contexts. Lastly, sensitivity to change in the current work is restricted to within‐subject differences between OFF and ON levodopa states and does not capture longitudinal progression of FOG over time. As such, the natural evolution of FOG severity, as well as responsiveness to other clinically relevant interventions—including alternative pharmacological treatments, rehabilitative physical therapy approaches, or emerging cueing technologies—remains undetermined. Nonetheless, this work lays the foundation for advancing therapeutic development in FOG to evaluate longitudinal and intervention‐specific sensitivity.

Qualitative analysis of patient interviews provides thorough insight into the impact of FOG in patients' daily lives, as well as how well the PRO reflects this impact. Aligning observable clinical assessment with patient‐perceived impact is critical to ensuring that measured changes in FOG severity reflect outcomes that are meaningful for the lived experience. Building on this foundation, future work will focus on further refinement of the GP‐FOG and FOG‐QOL using advanced psychometric approaches, including Rasch analysis, with the aim of improving measurement precision and reducing instrument length while preserving content validity. In addition, the integration of IMUs within the current protocol provides a unique opportunity to support future development of automated smart‐scoring of the GP‐FOG and individual FOG episodes. Algorithms capable of estimating FOG severity without the need for trained raters or annotators would enhance the scalability and accessibility of assessment. Furthermore, the inclusion of free‐living motion sensor data within this protocol enables future evaluation of the ecological validity of these tools and thereby the extent to which the in‐clinic assessments reflect real‐world FOG experiences. Finally, although the current cohort is representative of individuals with FOG in PD, further validation in additional subgroups, such as those with implanted deep‐brain stimulus devices and those using a walking aid, will be necessary to expand upon the GP‐FOG's use.

Collectively, these efforts will support the continued evolution of standardized, sensitive, and meaningful outcome measures, ultimately facilitating the development and evaluation of effective interventions for freezing of gait. The outcomes from the FOG‐COA project will make the next critical step forward in advancing FOG evaluation, enabling future clinical trials.

## Author Contributions

**Erika E. Howe:** methodology, data curation, investigation, validation, formal analysis, visualization, project administration, writing – original draft, writing – review and editing. **Pieter Ginis:** writing – review and editing, project administration, software, data curation, validation, conceptualization, investigation, funding acquisition, methodology. **Kaylena A. Ehgoetz Martens:** writing – review and editing, conceptualization, funding acquisition, supervision, formal analysis, methodology, project administration, validation, resources, investigation, writing – original draft. **Moran Gilat:** writing – review and editing, conceptualization, funding acquisition, supervision, methodology, resources, investigation, validation, project administration. **Martina Mancini:** conceptualization, funding acquisition, writing – review and editing, supervision, methodology, software, investigation, resources, project administration, validation. **J. Lucas Mckay:** conceptualization, investigation, funding acquisition, writing – review and editing, validation, methodology, software, project administration, supervision, resources. **Jorik Nonnekes:** conceptualization, funding acquisition, writing – review and editing, supervision, methodology, resources, project administration, investigation, validation. **Jennifer Mammen:** writing – review and editing, visualization, formal analysis, methodology, project administration, investigation, data curation, supervision, conceptualization, funding acquisition. **Jamie L. Adams:** conceptualization, investigation, funding acquisition, methodology, validation, writing – review and editing, project administration, resources, supervision. **David Cella:** conceptualization, methodology, writing – review and editing. **Alice Nieuwboer:** conceptualization, writing – review and editing, funding acquisition, supervision, methodology, resources, investigation, validation. **Fay Horak:** conceptualization, funding acquisition, writing – review and editing, supervision, methodology, resources, project administration, formal analysis, investigation, validation. **Simon J. G. Lewis:** conceptualization, investigation, funding acquisition, methodology, validation, writing – review and editing, project administration, supervision, resources. **Jeffrey M. Hausdorff:** supervision, project administration, writing – review and editing, funding acquisition, conceptualization, methodology, resources, investigation, validation, formal analysis.

## Funding

Michael J. Fox Foundation (MJFF‐024177). J.L.M. was supported in part by the McCamish Parkinson's Disease Innovation Program at Georgia Tech and Emory University.

## Disclosure

This work was funded by the Michael J. Fox Foundation for Parkinson's Research (MJFF‐024177). Dr. Adams has consulted for and received research funding from the Michael J. Fox Foundation for Parkinson's Research. Dr. Horak received institutional research funding from NIH, DOD, Michael J. Fox Foundation. Dr. Hausdorff's institution received funding from NIH and the Michael J. Fox Foundation for Parkinson's Research. Dr. Mancini received funding from NIH, Michael J. Fox Foundation for Parkinson's Research, and OHSU Parkinson Center. Dr. Howe received funding from Parkinson's Canada. Dr. Nonnekes was supported by the Michael J. Fox Foundation and has received funding from ZonMW, Gossweiler Foundation, Ataxie Vereniging and ERN‐RND outside the submitted work. Dr. Ehgoetz Martens has received research funding from Parkinson Canada, NSERC, Weston Family Foundation, Michael J. Fox Foundation, Canada Foundation for Innovation, CIHR Canada Research Chairs program, and NIH. Dr. Cella has received research funding to his institution from NIH, Michael J. Fox Foundation, and the National Quality Foundation. He has also received consulting fees from Oregon Health and Science University. Dr. Lewis is supported by a National Health and Medical Research Council Leadership Fellowship (1195830) and has received research funding from the Michael J. Fox Foundation and the Australian Research Council. J.L.M., A.N., and M.G. have nothing to report.

## Conflicts of Interest

Dr. Horak has been employed by Clario Inc., who manufactures and distributes the Oal inertial sensors used in this study. This potential conflicts of interest is reviewed by OHSU.

Dr. Ginis is an employee of Clario, part of Thermo Fisher Scientific, who manufactures and distributes the OPAL inertial sensors used in this study. He joined Clario after this study was already designed and started.

All other authors declare no conflicts of interest.

## Supporting information


**Data S1:** Patient meaningfulness survey.
**Data S2:** Activity diary for home‐monitoring.
**Data S3:** Pre‐interview survey.

## Data Availability

Not applicable. This is a protocol paper to propose the methods for the validation of the FOG‐COAs; the validation work is ongoing.
