# Supplementary material for: The FOG‐COA Protocol: Validation of Freezing of Gait Clinical Outcomes Assessments in Parkinson's Disease
Source: Eur J Neurosci. 2026 Oct 17;64(8):e70701. doi: 10.1111/ejn.70701 (PMC13675721; doi:10.1111/ejn.70701)
Supplement: Supplementary file 1 [file EJN-64-0-s001.docx]

# APPENDICES

## Supplement I: Patient Meaningfulness Survey

**Objectives**

During the FOG-COA study, participants will be asked to describe the severity. impact and limitations caused by FOG. Participants will also be asked to describe any changes in FOG severity occurring between the OFF and ON state testing, as well as the everyday relevance of each task conducted in the clinically rated outcome (ClinRO). Finally, participants will be asked if there are any tasks representing impacts of their FOG that are missing from the battery. These items will help ensure that the final COA set includes measures that are meaningful to individuals regarding their physical function.

**How do I administer this survey?**

All of these items will be administered verbally in an interview format by the study staff at the first study visit. Participant’s responses will be recorded by study staff in REDCap or on paper for later data entry. Many items will ask how the patient for a rating. When there is an open ended question, please encourage participants to fully elaborate on their responses.

**Prior to GP-FOG Assessment (in the OFF state), please ask the patient to rate each question:**

1. How severe is your FOG? ______

(0-not at all severe; 10- extremely severe) NA = not applicable

2. How important is FOG to you personally (even if you don’t currently experience FOG)? _____

(0-not at all important; 10- extremely important)

3. How severe is your falling? ______

(0-not at all severe; 10- extremely severe)

4. How important is falling to you personally (even if you don’t currently experience falling)? _____

(0-not at all important; 10- extremely important)

*Note: Importance refers to how ‘important’ FOG symptom is, EVEN IF YOU DON’T CURRENTLY EXPERIENCE FOG NOW, whereas ‘bothersome’ refers to both experiencing and thinking or worrying about FOG and the burden that is associated with these experiences IN DAILY LIFE.*

**After each trial of the GP-FOG (in the OFF state), please ask the patient to rate each question:**

In cases where the participant was unable or unwilling to attempt a task, please select the response option in the corresponding column. Study staff will indicate the reason for missing data either using standard response options (e.g., participant refused or declined, lack of time, safety concerns, etc) or in cases where the reason for missing data is not accurately captured by the standard response options, by using free text.

| Trial | ***Did you have difficulty with performing this test?***  *(0-no difficulty;*  *10 – severe difficulty)* | ***How severe was your FOG during that task?***  (0-not at all severe; 10- extremely severe)  NA = not applicable | ***Do you feel the task is a good test of your FOG?***  (0-not a good test at all; 10- excellent)  NA = not applicable | ***How does the task relate to things you do in daily life?***  (0-does not relate; 10- relates very well) | ***Unable to attempt*** | ***Unwilling*** |
| --- | --- | --- | --- | --- | --- | --- |
| Walk |  |  |  |  |  |  |
| DT Walk |  |  |  |  |  |  |
| Carrying |  |  |  |  |  |  |
| Turn |  |  |  |  |  |  |
| DT Turn |  |  |  |  |  |  |
| Box Shuffle |  |  |  |  |  |  |
| Box Agility |  |  |  |  |  |  |
| Doorway |  |  |  |  |  |  |

**After the GP-FOG Assessment is completed (in the OFF state), please ask the patient:**

5. How severe was your FOG in the OFF state? ______

(0-not at all severe; 10- extremely severe)

6. Do you feel like that whole test was a good test of FOG? ______

(0-not a good test at all; 10- excellent)

**After the GP-FOG Assessment is completed (in the ON state), please ask the patient:**

7. How severe was your FOG in the ON state? ______

(0-not at all severe; 10- extremely severe)

8. Do you feel like that whole test was a good test of FOG? ______

(0-not a good test at all; 10- excellent)

9. Compared to your OFF today, how would you rate the change in your FOG?

☐ Much better FOG since I started this study

☐ A little better FOG since I started this study

☐ No change in FOG that is meaningful to me since I started this study

☐ A little worse FOG since I started this study

☐ Much worse FOG since I started this study

**I would like you to consider these three questions between now and the next visit, and when you return, I will ask you these questions again:**

10. Thinking about the things you do in your daily life, from when you wake up to when you go to sleep at night, what are some things you do that are important to you that require the tasks we just completed (e.g. turning on the spot; walking through a doorway; shuffling around a box; concentrating on another task while walking/turning/negotiating a door)?
11. Does the difficulty of these tasks match the difficulty of daily activities that you mentioned?
12. Thinking about the things you do in daily life, what are some activities that are impacted by FOG that are not reflected in the tasks we just completed?

## Supplement II: Activity Diary for Home-Monitoring

## Supplement III: Pre-Interview Survey

Section 1: Symptom Survey

Do you experience any of the following symptoms: (Please check YES/NO/unsure)

| **MOVEMENT CHANGES** | **THINKING CHANGES** | **SPEECH CHANGES** |
| --- | --- | --- |
| Shaking (tremor) | Changes in thinking | Changes in your speech |
| Sense of internal tremor | Difficulty concentrating on things | Changes in your voice |
| Trouble with hand dexterity | Slower thought process | Quiet speech |
| Changes in handwriting | Difficulty remembering things | Monotone speech |
| Slower movements overall | Difficulty coming up with words in a conversation | Difficulty pronouncing things |
| Changes in your balance | Trouble making decisions |  |
| Changes to your walking | Mental fogginess | **MOOD CHANGES** |
| Freezing | Trouble with depth perception | Changes in mood |
| Increased stiffness in muscles/joints | Getting lost or trouble finding way | Feeling more anxious |
| Stooping posture |  | Feeling more depressed |
| Decreased range of motion | **SLEEP CHANGES** | Less interested in things |
| Loss of general coordination | Trouble getting to sleep | More easily frustrated |
| Decreased arm swing when walking | Trouble staying asleep | More negative feelings/emotions |
| Decreased facial expressiveness | Poor sleep quality | Decreased pleasure in things |
| Muscle cramping or spasming | Active dreaming (kicking, shouting) | Mood swings |
| Restless legs | Excessive daytime sleepiness | Impulsive behaviors |
| Twitching |  |  |
| Dyskinesia |  | Changes in your personality |
| **DIGESTIVE CHANGES** | **URINARY CHANGES** | **SENSORY CHANGES** |
| Excess saliva | Urinary problems | Changes in vision |
| Dry mouth | Frequent urination | Changes in smell |
| Issues with swallowing pills or food | Getting up often at night to urinate | Changes in sense of taste |
| Choking when eating or drinking | Urinary incontinence | Changes in hearing |
| Constipation |  | Changes in your skin sensation |
| Appetite changes | **SEXUAL CHANGES** | Increased pain |
| **OTHER CHANGES** | Trouble achieving orgasm | Fatigue or lack of energy |
| Low blood pressure | Loss of sexual interest | Shortness of breath |
| Feeling lightheaded or dizzy | Erectile dysfunction (men) | Feeling of weakness |
|  | Vaginal dryness (female) | Altered temperature tolerance |

Section 2. Bothersome symptoms

1. Please describe the PD symptoms that are most bothersome to you. If possible, list these one at a time. Feel free to say what you think has caused the changes and to describe the ways these affect you. We will talk about these more during the interview.

(Open response)

2. Of the changes you described, **which ones are most important to you** and why?

(Open response)

3. Are any of the symptoms you experience important, but not yet bothersome to you?

(Open response)
